# Case Report: Traumatic Stress and Developmental Regression: An Unintended Consequence of Complex Cardiac Care

**DOI:** 10.3389/fped.2021.790066

**Published:** 2021-12-24

**Authors:** Claire M. Dahl, Maria Kroupina, Sameh M. Said, Arif Somani

**Affiliations:** ^1^Department of Pediatrics, University of Minnesota, Minneapolis, MN, United States; ^2^Division of Pediatric Cardiovascular Surgery, Masonic Children's Hospital, Minneapolis, MN, United States; ^3^Department of Surgery, University of Minnesota Medical School, Minneapolis, MN, United States

**Keywords:** case report, mental health, pediatric development, critical care, pediatric intensive care unit

## Abstract

This brief case report outlines a novel approach to supporting the development of a pediatric complex cardiac care patient. Patient X is a 19-month old patient who spent 5.5 months in hospital and underwent multiple surgeries including heart transplantation. This case report explores the impacts of his condition and care on his development and family functioning within the framework of an integrated care model. This case report is uniquely complimented by outpatient neurodevelopmental follow up, dyadic trauma-informed intervention and use of telemedicine allowing for a deeper understanding of the family adaptation that provide novel insight into long-term trajectory beyond discharge. Throughout care Patient X met criteria for both a traumatic stress disorder and global developmental delay. This case study highlights the threat complex care poses to neurodevelopment, pediatric mental health and family dynamics as well as opportunities for intervention.

## Introduction

Hospitalization in childhood disrupts normative developmental processes and induces significant stressors on both children and their parents. Necessary but uncomfortable therapeutic interventions, the loss of a familiar and predictable environment, and the limited opportunities for exploration all contribute to an abnormal developmental environment. Separation from primary caregivers further exacerbates possible threats to normal development in this environment. An optimal caregiver-child relationship is one of the strongest protective factors against comprised development and detrimental psychological and physical consequences (1–3). The following case report exemplifies the implications of inpatient stress on long-term outpatient child development. This case is uniquely complimenting by long-term outpatient neurodevelopmental and mental health follow-up and interventions allowing for perspectives in post-discharge functioning. It is instructive that the stress and challenges associated with acute and chronic hospitalization can impact the patient's developmental trajectory as well as the parent-child relationship. Given the lack of a standardized approach to mitigate such challenges, opportunities for early recognition are identified in order that intervention maybe leveraged in the inpatient environment and post-discharge to optimize outcomes.

## Case Description

**Figure 1:**
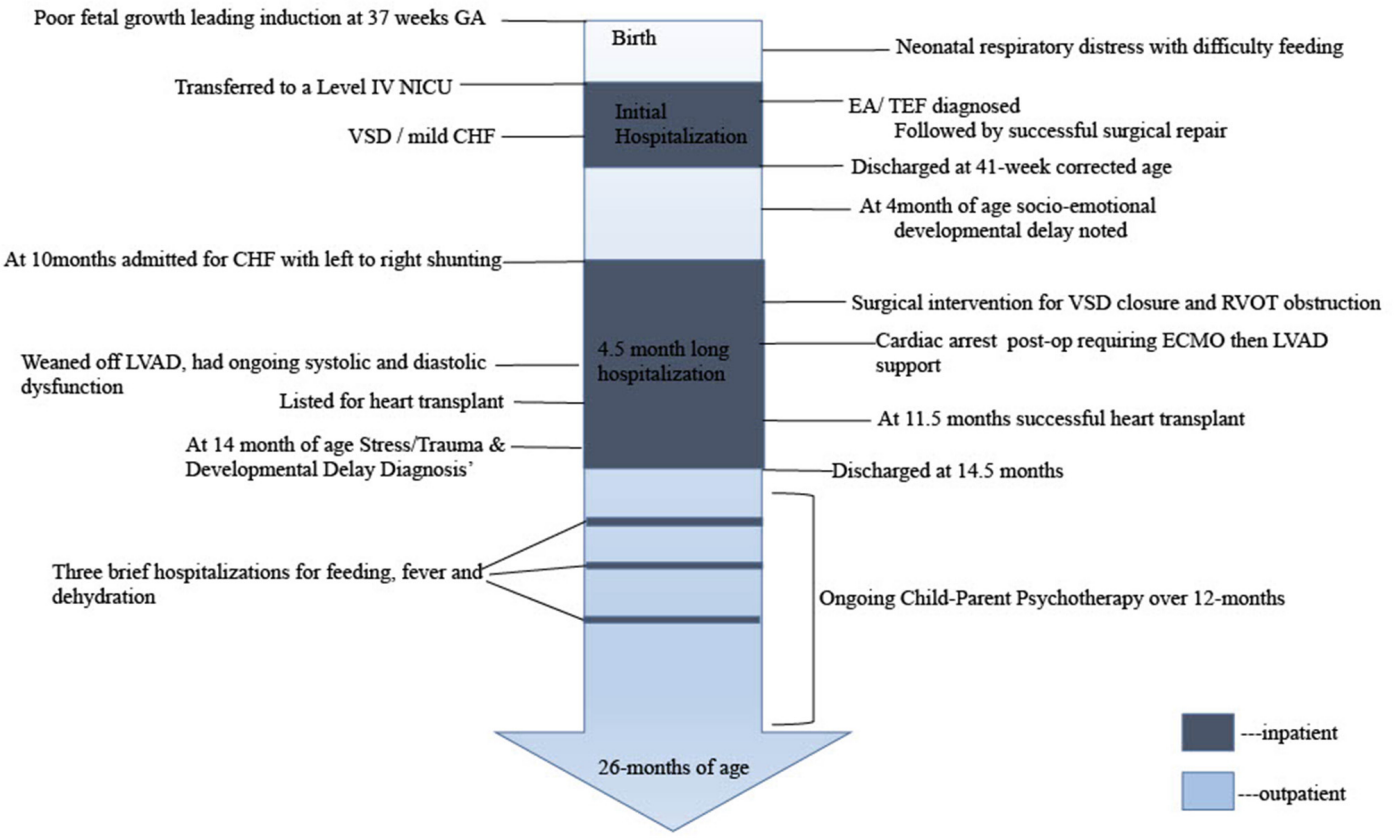
Timeline of care.

The parents of this patient provided informed consent for data collection, chart review and presentation of findings. A timeline of care is outlined in Figure 1.

### Medical History

P is a 19-month-old, ex-37 week, small for gestational age male; with a birth weight of 2,220 grams. Labor was induced due to poor fetal growth. Although initial APGAR scores were reassuring; shortly after birth, difficulty with feeds and respiratory distress necessitated transfer to a Level IV NICU for suspected esophageal atresia (EA) with tracheoesophageal fistula (TEF). This was confirmed and surgical repair was undertaken without complications. Post-natal echocardiogram demonstrated large perimembranous ventricular septal defect (VSD) and mild left ventricular enlargement managed with furosemide. P was discharged from the NICU at 41-weeks corrected gestational age, and was compliant with follow-up appointments.

**Figure 2:**
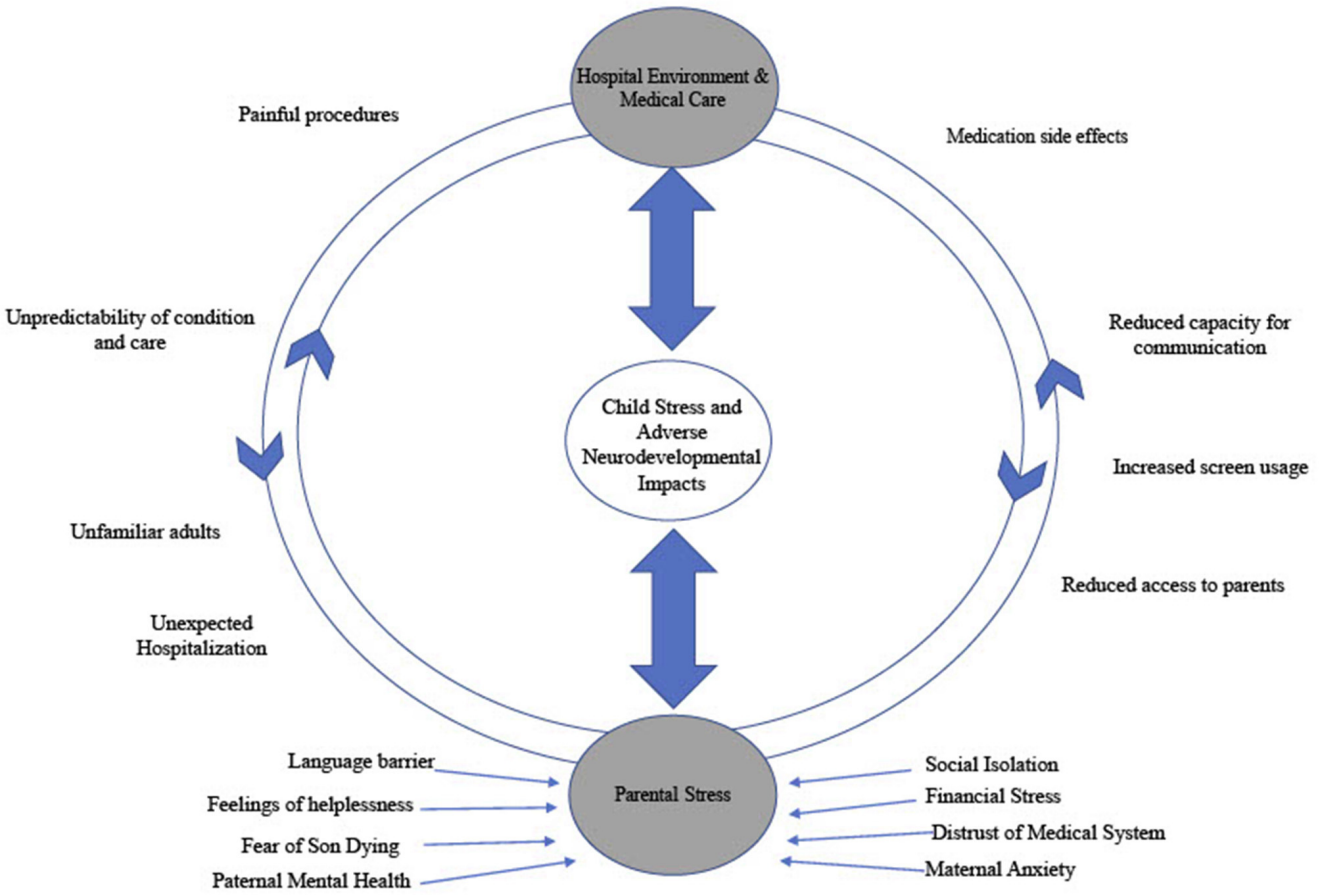
Impact of stressors on Neurodevelopment in hospitalized children.

At his 4-month follow-up appointment the patient was seen by an early childhood mental health clinician (PhD, LP) where a slight delay in social-emotional development was noted via Ages and Stages Developmental Questionnaire (4). The patient displayed age-appropriate social behavior (smiles, responding to voices) but was behind expectations on self-regulatory skills (self-soothing). Identified family stressors included strained financial resources, limited access to care due to rural residence, and pending maternal immigration status. Please see Figure 2 for a summary of intersecting stressors influencing and compounding clinical presentation. Referral was made for local state supportive intervention services.

At 10 months of age an abnormal ECHO demonstrated significant large left-to-right shunt secondary to a large VSD, and right ventricular outflow tract (RVOT) obstruction and a subaortic gradient, requiring surgical resection of right ventricular outflow tract muscle bundles and of the subaortic membrane and VSD closure. This procedure (on 7/15/20) was uncomplicated with good results on postoperative transesophageal echocardiogram. However, his post-op course was complicated by bradycardic cardiac arrest of unknown etiology on the first postoperative night requiring extracorporeal membrane oxygenator (ECMO) support, and thereafter left ventricular assist device (LVAD) support. He was able to be weaned off the LVAD but had ongoing severe systolic and diastolic dysfunction requiring inotropes and ongoing mechanical ventilation. His course was complicated by a stable subdural hemorrhage with mirco-hemorrhages throughout the cerebral, midbrain and cerebellum. He was listed for heart transplant. Decompensation led to a Berlin LVAD placement on 8/26/20, followed by heart transplant on 8/30. Post-transplant imaging displayed global cerebral volume loss with confluent hypodensity of the periventricular white matter. He required prolonged post-transplant hospitalization given his deconditioning, oral aversion, aspiration risk and required supportive care. He was discharged on 11/10/2020 at 14.5 months of age. He presented back to the hospital for three brief stays due to dehydration, fever, and feeding intolerance, respectively. Overall, this patient spent ~5.5 months in inpatient care.

During hospitalization, a childhood mental health clinician was consulted due to poor parental coping, post-transplant concerns of developmental regression and an abnormal stress response. At this time the family reported that they had been unable to fulfill the referral for supportive services in their area that had been recommended at 4-months. A detailed developmental assessment, including parent interview, observations of the child and the parent-child dyad and consultation with medical team was conducted. This assessment revealed increased social withdrawal (interacting solely with his iPad and not parents or social partners), reduced positive affect (decrease in positive displays of emotions), fearful behaviors (withdraw and dysregulation in response to medical staff or new people), hypervigilance (constant awareness of changes in the environment) and difficulties with concentration (he could only display maintained attention with the iPad) and disordered sleep—all manifestations that are characteristic of Post-Traumatic Stress Disorder (PTSD). However, he did not meet full PTSD criteria (namely re-experiencing trauma and avoidance of reminders), and thus, was diagnosed with Other Trauma, Stress and Deprivation Disorder of Early Childhood. The slight delay in social and emotional development that was noted at 4 months had progressed to a diagnosis of global developmental delay by 14 months of age due to the absence of social, cognitive, motor and language skills.

### Family Functioning at Second Hospitalization

The consultation with this family at 10.5 month of age illustrates challenges faced by the family and possible gaps in the system. Paternal mental health challenges, aversions to hospital visits, maternal anxiety and a distrust of medical system were noted; all complicated by cultural and language barriers. This is also encapsulated in Figure 2. The clinician noted that the patient rarely looked to parents for emotional support or security. The child was utilizing a screen for regulation rather than reaching out to his parents for support. This accentuated their feelings of helplessness and made them feel dispensable in his care; further promoting a cycle of isolation.

Due to symptoms of trauma in this patient and the level of parental distress the clinician recommended trauma-informed dyadic intervention (see Table 1). Unfortunately, there are currently no standardized dyadic psychological interventions formalized for children in the critical care environment. The clinician saw the family approximately twice per month while the patient was still in the hospital, offering supportive resources for the parents and suggestions on how to enhance interactions. Both parents were receptive to these services.

**Table 1:** Trauma-informed relational interventions.

**Name**	**Populations**	**Evidence based for:**	**Research outcomes**
Child-parent psychotherapy (CPP)	Children 0–6	Trauma histories, behavioral challenges or transitions in caregivers (8)	Higher quality attachment relationships, positive impacts on cognitive development, reduction of PTSD symptoms & mental health diagnosis (8)
Attachment bio-behavioral catch-up (ABC)	Children 0–5	Preventative intervention for high risk infants/children (foster care etc.), high risk parents (addiction history) (22)	Increase in parent sensitivity, diurnal cortisol pattern, higher quality attachment relationships, increased inhibitory control and cognitive flexibility (22)
Parent child interaction therapy (PCIT)	Children 0–3	Oppositional behaviors, and behavioral challenges (23)	Improve parental stress and parenting skills, reduce pediatric internalizing and externalizing problemts (23)
Video feedback to promote positive parenting and IMH	Children 0–8	High risk parents (poverty, maltreatment etc.), high risk children (externalizing challenges, adopted, ASD) (24)	Increase in positive parenting, reductions in externalizing behaviors, improved quality childcare (24)

### Outpatient Course

At the point of discharge, the family began trauma informed dyadic intervention with the same clinician primarily via telemedicine (and which continued over the next 12 months). The patient continued to show an abnormal stress response with hypervigilance, hyperactivity or complete passivity. This is noteworthy as the child attended these visits with both parents from his home, where environmental components should be supportive. Once an intensive stressor is removed (such as the hospital environment) ideally children are expected to signal their distress to primary caregivers and seek comfort (5). The continuation of abnormal stress responses in the absence of the inpatient environment and in the presence of his parents was of concern; capacity to find comfort in a caregiver when stressed is an important developmental skill to reduce emotional and physiological distress (2, 6, 7). The family is now engaging in weekly therapy with the clinician, following the Parent-Child Psychotherapy intervention model (8). Of note, the Covid-19 pandemic necessitated weekly therapy appointments via a tele-health model. As restrictions were lifted the goal was to alternate therapy sessions weekly between in-person and tele-health. While disadvantages to tele-medicine have been reported (9), the flexibility of telemedicine allowed for higher compliance with scheduled visits and a lower likelihood of cancellation due to maternal anxiety, family schedule or illness. Telemedicine may have a particular advantage in this population of vulnerable children as both the child and the parents may be hesitant to return to the hospital environment given associated triggers or stressors. Continued work to attenuate such hospital avoidance is vital to allow for a full in-person developmental assessments to optimize cognitive and social functioning. Advances have been made in helping the patient's parents identify his irregular indicators of stress and maladaptive responses as well as how they may best respond. Through the development of this ongoing therapeutic process a deeper understanding of the parent's perspective, emotions and experience was gained. Further goals are centered around decreasing parental distress and enhancing the family's ability to engage in synchronous dyadic interactions.

## Discussion

This case study highlights the threat hospitalization and complex care pose to neurodevelopment and pediatric mental health as well as opportunities for intervention. When the family was seen at their four-month follow up appointment they were undoubtedly experiencing stress and social isolation, but it was manageable. However, they did not connect with supportive services and were unable to develop effective coping strategies. In the absence of these strategies and with the additional stressors of re-admission and cardiac transplantation they struggled to regulate their own stress and were unable to help co-regulate their son's stress. Furthermore, despite what many medical professionals may hope, the child's stress and trauma symptoms did not resolve upon discharge and removal from a “stressful environment.” Indeed, ongoing and unmanaged stressors continued to put his development at risk. Effective consultation and intervention are needed both in the inpatient stay and upon discharge in order to ensure that parents and their children do not continue to struggle with concerning mental health symptoms.

While the themes in this case are commonly identified in complex chronic hospitalized pediatric patients, the specific diagnosis of Other Trauma, Stress Disorder of Early Childhood is both a significant and escalating factor in the assessment and care of this patient, and is indicative of detrimental implications in his post-discharge course. These maladaptive stress responses, even in the home environment, is a marker of the severity of his accumulated insults and may serve as a poor prognostic indicator in terms of future development and adaption in the absence of intervention (2, 3, 6). It is important to note that there is no clear etiologic factor as to what element of critical care or hospitalization primarily impact development, and such an answer likely does not exit. A variety of components of critical cardiac care can have an adverse impact on neurological and neuropsychological development including, surgery, anesthetics and bypass interventions (10–13). Additionally, children born with complex congenital heart lesions are frequently born with brain immaturity and with ongoing relative hypoxemia (14–16). Teasing apart the impact of neuro-organic insults as opposed to stress and environmental factors is a clear limitation in this case study. These organic factors alone do not fully explain the developmental delay seen in this patient or many of those who experienced similar care. Additionally, such neuronal insults are not commonly seen to elicit the trauma response seen in this patient. Specific factors (over both the inpatient course and in the post-discharge home environment) that contribute to adversely impacting neurodevelopment beyond the neuro-organic insults are outlined in Figure 2. This inability to tease apart and quantify specific elements of an experience is a challenge that has been noted consistently in the developmental trauma literature (17, 18).

Early childhood is a period of in which neurodevelopment is uniquely vulnerable to insult. The brain utilizes early plasticity to promote learning and adaptation, however this plasticity also leaves the brain open to impacts that can cascade through later development. A child's developing stress system was not designed for chronic or incredibly heightened activation Extreme stress in the absence of a safe and predictable caregiving relationship can lead to an inability to regulate the developing stress system (6). A dysregulated stress system may have adverse impacts on brain, immune, hormonal and behavioral development throughout the lifespan (1, 19, 20). Given the plastic nature of early brain development young children can also display outstanding capacity for recovery and resilience (21). Evidence based dyadic interventions (as described in Table 1) show promise in reducing children's behavioral manifestations of past trauma, promoting a more well-regulated physiological stress system and enhancing capacity for learning (22–24). Selecting the dyadic relationships as a point of intervention offers opportunity to support both young patients and their parents. The trauma and stressed faced by children experiencing complex critical care cannot be completely removed; however, trauma informed dyadic intervention can alleviate the toll of this experience and attenuate adverse sequelae.

While developmental concerns are acknowledged by medical professions in post-cardiac surgery populations, the severity of the developmental delay and the prolonged symptoms of post-traumatic stress disorder in this case are both significant and novel enough to warrant discussion. The possible impact on the child's development due to long hospitalization related to complex and repeat cardiac surgical procedures should be disclosed as part of the consent process. Furthermore, despite a level of acknowledgment among medical care providers there is little in the literature on the psychological and developmental consequences of complex cardiac care. Limited research suggests that childhood hospitalization is correlated with learning delays (25), heightened physiological stress response (26) and/or increased levels of psychosocial stress (27). Further research is needed to appropriately understand and address the needs of this vulnerable population. In the field overall, *long-term* neurodevelopmental follow-up is not the current standard of care. However, patients may undoubtedly benefit from developmental follow-up and care post-surgery and hospitalization. The program through which patient X was managed is unique in its capacity to meet families during hospitalization and provide support post-discharge. During hospitalization the primary focus is on the child's physical health and survival; however, failure to address mental health concerns in these families will place the child's long-term development at risk. As such, early identification and intervention with long-term follow up should be an ideal model for care for these families.

## Summary

This case highlights the potential impact of intense and complex cardiac care on neurodevelopment, pediatric mental health and family dynamics, as well as novel intervention mechanisms.

## Data Availability Statement

The original contributions presented in the study are included in the article/supplementary material, further inquiries can be directed to the corresponding author/s.

## Author Contributions

CD conceptualized and drafted the initial manuscript and additionally contributed critical revisions. MK played a vital role in conceptualization, patient identification, and critical manuscript drafting and revision. SS substantially contributed to conceptualization and critical manuscript revision. AS made substantial contributions to conceptualization, patient identification, manuscript drafting, and critical revision. All authors approved the final manuscript as submitted and agree to be accountable for all aspects of the work.

## Conflict of Interest

SS is a consultant for Stryker and Cryolife. The remaining authors declare that the research was conducted in the absence of any commercial or financial relationships that could be construed as a potential conflict of interest.

## Publisher's Note

All claims expressed in this article are solely those of the authors and do not necessarily represent those of their affiliated organizations, or those of the publisher, the editors and the reviewers. Any product that may be evaluated in this article, or claim that may be made by its manufacturer, is not guaranteed or endorsed by the publisher.

## Abbreviations

EA: esophageal atresia
TEF: tracheoesophageal fistula
VSD: ventricular septal defect
NICU: neonatal intensive care unit
RVOT: right ventricular outflow tract
ECMO: extracorporeal membrane oxygenator
LVAD: left ventricular assist device
PTSD: posttraumatic stress disorder.
